# Prospective Analysis of Day-One Postoperative MRI Following Cervical Decompression for Cervical Myelopathy: Insights into Residual Compression and Signal Changes

**DOI:** 10.7759/cureus.92699

**Published:** 2025-09-19

**Authors:** Bharat R Dave, Abhijith Anil, Sandesh Agrawal, Mahesh Sagar, Mirant B Dave, Mikeson Panthackel, Shivanand C Mayi, Ravi Ranjan Rai, Ajay Krishnan, Arjit Vashishtha, Amritesh Singh

**Affiliations:** 1 Spine Surgery, Stavya Spine Hospital and Research Institute, Ahmedabad, IND; 2 Orthopaedics/Spine Surgery, Pushpagiri Institute of Medical Sciences and Research Centre, Thiruvalla, IND; 3 Spine Surgery, Bhavnagar Institute of Medical Science (BIMS), Bhavnagar, IND; 4 Orthopaedics, Geetanjali Medical College and Hospital, Udaipur, IND; 5 Orthopaedics, University College of Medical Sciences, Guru Teg Bahadur Hospital, Delhi, IND

**Keywords:** cervical spondylotic myelopathy, chen’s grading, hoffman sign, magnetic resonance imaging (mri), postoperative

## Abstract

Background

Cervical myelopathy is a progressive condition caused by spinal cord compression, often requiring timely surgical decompression. Although clinical improvement is generally expected after surgery, the role of immediate postoperative magnetic resonance imaging (MRI) in assessing decompression adequacy and predicting neurological recovery remains underexplored. This study examines the value of early postoperative MRI in identifying residual stenosis, missed levels, and intramedullary signal changes.

Methods

A total of 181 patients who underwent cervical decompression surgery for cervical spondylotic myelopathy (CSM) were prospectively included. Depending on the stenosis level, patients underwent posterior laminectomy with or without fusion, or anterior cervical discectomy/corpectomy. Preoperative and immediate postoperative MRI (within 24 hours) were evaluated for decompressed levels, residual stenosis, missed levels, and intramedullary T2 signal changes, graded using Chen’s grading. All MRIs were independently reviewed by a neuroradiologist and cross-checked by the spine surgeon, both blinded to clinical status. Statistical analysis was performed using a paired t-test.

Results

Of the 181 patients, 125 had CSM and 40 had ossification of the posterior longitudinal ligament. The posterior approach was used in most cases due to multilevel involvement. Postoperative MRI showed persistent stenosis in seven patients, with early detection enabling timely intervention. Signal intensity changes were observed in 139 patients, with increased size and clarity in 121 cases. These changes in intramedullary signal intensity were statistically significant (p < 0.001). Four cases of missed-level decompression were identified and corrected during the same admission. Non-compressive hematomas were noted in 14 patients, and cerebrospinal fluid (CSF) collections in 12. In one patient, immediate postoperative MRI revealed persistent compression at the C1 level after C2-C7 laminectomy, requiring reoperation for adequate decompression.

Conclusion

Immediate postoperative MRI is valuable for confirming decompression adequacy, identifying residual compression, and detecting complications such as CSF leaks and hematomas. The transient increase in intramedullary signal changes underscores the need for further research on their progression and correlation with long-term neurological recovery. Routine postoperative MRI screening, particularly with limited sagittal sequences, can enhance patient safety, prevent delayed interventions, and potentially improve surgical outcomes. Future studies should explore long-term signal evolution and its relationship to clinical recovery, especially in thoracic myelopathy, where evidence remains limited.

## Introduction

Cervical spondylotic myelopathy (CSM) is the most common cause of spinal cord dysfunction worldwide, presenting with symptoms such as gait instability, bladder dysfunction, and fine motor deficits [1]. These manifestations result from degenerative changes in the cervical spine that lead to spinal cord ischemia and axonal injury [2]. Advanced cases often require surgical decompression, usually with fusion. In the United States, the number of surgeries for cervical spine degeneration has risen significantly, with anterior cervical fusions accounting for 80% of procedures [3]. By 2009, the annual number of such surgeries reached 186,679, with reoperations contributing to as much as 2% of the total costs, highlighting the growing economic burden [3,4].

Although thoracic myelopathy is less common, it presents unique challenges, often caused by ossification of the posterior longitudinal ligament, ligamentum flavum, or disc herniations. Surgery rates are considerably lower than those for CSM. Postoperative complications such as epidural hematomas, inadequate decompression, and cerebrospinal fluid (CSF) leaks require prompt recognition and management. Imaging plays a key role in detecting these complications and guiding treatment. Among available modalities, magnetic resonance imaging (MRI) remains the gold standard for identifying infections, hematomas, and nerve root compression. Advanced techniques, including fat-suppressed and contrast-enhanced sequences, further improve diagnostic accuracy despite artifacts from surgical implants [5].

Surgical errors, such as wrong-level spine surgery, add another layer of complexity. Studies show that up to 67% of spine surgeons encounter this error during their careers, leading to significant clinical and legal consequences [6]. In patients with myelopathy, a missed or inadequate decompression can be especially serious, often resulting in irreversible neurological decline and poor postoperative recovery. To reduce these risks, our institution follows a protocol of performing immediate postoperative MRI within 24 hours of surgery, allowing early detection of complications and timely corrective intervention.

Routine postoperative MRI is not universally practiced due to high costs, longer scan times, and interpretative challenges from postoperative artifacts. However, recent literature supports its usefulness in the early postoperative period, even in the presence of metallic implants [7]. At our center, we use a streamlined protocol to perform rapid, focused MRI scans within 5-6 minutes, limited to sagittal views of the region of interest. These images are reviewed in real time by a radiologist specializing in spinal conditions, ensuring prompt identification of potential issues. Although increasingly adopted in lumbar discectomy and endoscopic spine procedures, the role of immediate postoperative MRI after decompression for cervical and thoracic myelopathy remains underexplored. The objective of this study was to evaluate the role of early postoperative MRI in patients undergoing cervical decompression for cervical myelopathy. Specifically, we aimed to assess (i) confirmation of adequate decompression, (ii) detection of missed levels, (iii) evolution of intramedullary signal changes, and (iv) identification of complications such as hematoma or CSF collections. While intraoperative radiographs and postoperative CT scans are valuable for confirming implant position and bony decompression, they cannot demonstrate residual cord compression or intramedullary signal alterations. MRI therefore provides unique, clinically relevant information in the immediate postoperative period. Many centers do not allow or advocate for day-one postoperative MRI due to financial constraints. At our center, we include in our routine charges and considere routine protocols for all myelopathy patients.

## Materials and methods

This was a prospective, single-center study conducted at Stavya Spine Hospital and Research Institute. A total of 181 patients who underwent surgery for cervical myelopathy over a one-year period were included. All patients had a postoperative MRI within 24 hours of cervical decompression surgery. Exclusion criteria were myelopathy due to acute trauma, patients who developed neurological deficits immediately after surgery requiring MRI on the same day, claustrophobic patients who declined postoperative MRI, patients with pacemakers, and those with myelopathy caused by intramedullary spinal cord tumors. The primary investigator evaluated all patients admitted for surgical management of cervical myelopathy. Demographic details, including age, sex, and etiology of myelopathy, were recorded. The number of compressed spinal levels and the severity of myelopathy were documented using Nurick grading and the modified Japanese Orthopaedic Association (mJOA) score. Preoperative MRI, particularly sagittal scans, was evaluated to document the number of compressed levels. Intramedullary signal intensity changes were assessed and graded using Chen’s classification, and the number of affected levels was recorded. Patients were reassessed on the first postoperative day, with the exact surgical procedure noted. Postoperative MRI was performed in all patients on day 1, limited to T2-weighted sagittal images of the operated region. Scans were obtained using a 1.5T Philips GE Multiva scanner (Philips Healthcare, Amsterdam, Netherlands) with a 16-element spine matrix coil. The scanner’s field of view extended up to 530 mm, and the average acquisition time for a single spinal region was 5-6 minutes.

All postoperative MRIs were independently reviewed by a fellowship-trained neuroradiologist experienced in spinal imaging and cross-checked by the primary investigator (spine surgeon). Reviewers were aware that the scans were postoperative but were blinded to patients’ clinical status and intraoperative findings to minimize bias. Intramedullary signal changes were graded using Chen’s classification, based on the intensity and clarity of hyperintensity on T2-weighted sagittal images. The cranial-caudal extent of signal changes and the number of affected levels were recorded. Adequacy of decompression was assessed on midsagittal T2-weighted images by evaluating restoration of the CSF column around the spinal cord. Missed levels were defined as preoperatively compressed segments not addressed during surgery. The presence of hematomas, CSF collections, or hemostatic materials such as Gelfoam was also documented. This standardized protocol ensured that postoperative MRI assessment was objective, reproducible, and consistent.

## Results

We reviewed postoperative MR images of 181 consecutive patients who underwent decompressive surgery for cervical myelopathy or combined cervical and dorsal myelopathy (tandem stenosis). The mean age was 59 years (±11.87), with 147 males and 34 females. Of these, 154 patients were operated on for cervical myelopathy alone, while 27 had both cervical and dorsal involvement. On average, each patient had stenosis at 2.98 levels (±1.43). Cervical spinal stenosis was graded according to Kang’s grading: 6 patients had Grade 1 stenosis, 56 had Grade 2, and 119 had Grade 3. 

Functionally, 12 patients were Nurick Grade 1, 11 were Grade 2, 85 were Grade 3, 39 were Grade 4, and 34 were Grade 5. The mean mJOA score among patients with cervical myelopathy was 11.93 (±2.65). Of the total cohort, 125 patients had CSM, 40 had ossification of the posterior longitudinal ligament causing neural compression, 6 had adjacent segment disease after prior fusion, and 7 had myelopathy secondary to atlantoaxial instability. Two patients developed myelopathy due to infectious pathology, and one had stenosis from ligamentum flavum hypertrophy. In terms of surgical management, 116 patients underwent cervical laminectomy alone, 31 underwent laminectomy with instrumented fusion, and 34 underwent anterior decompressive surgery.

**Figure 1:**
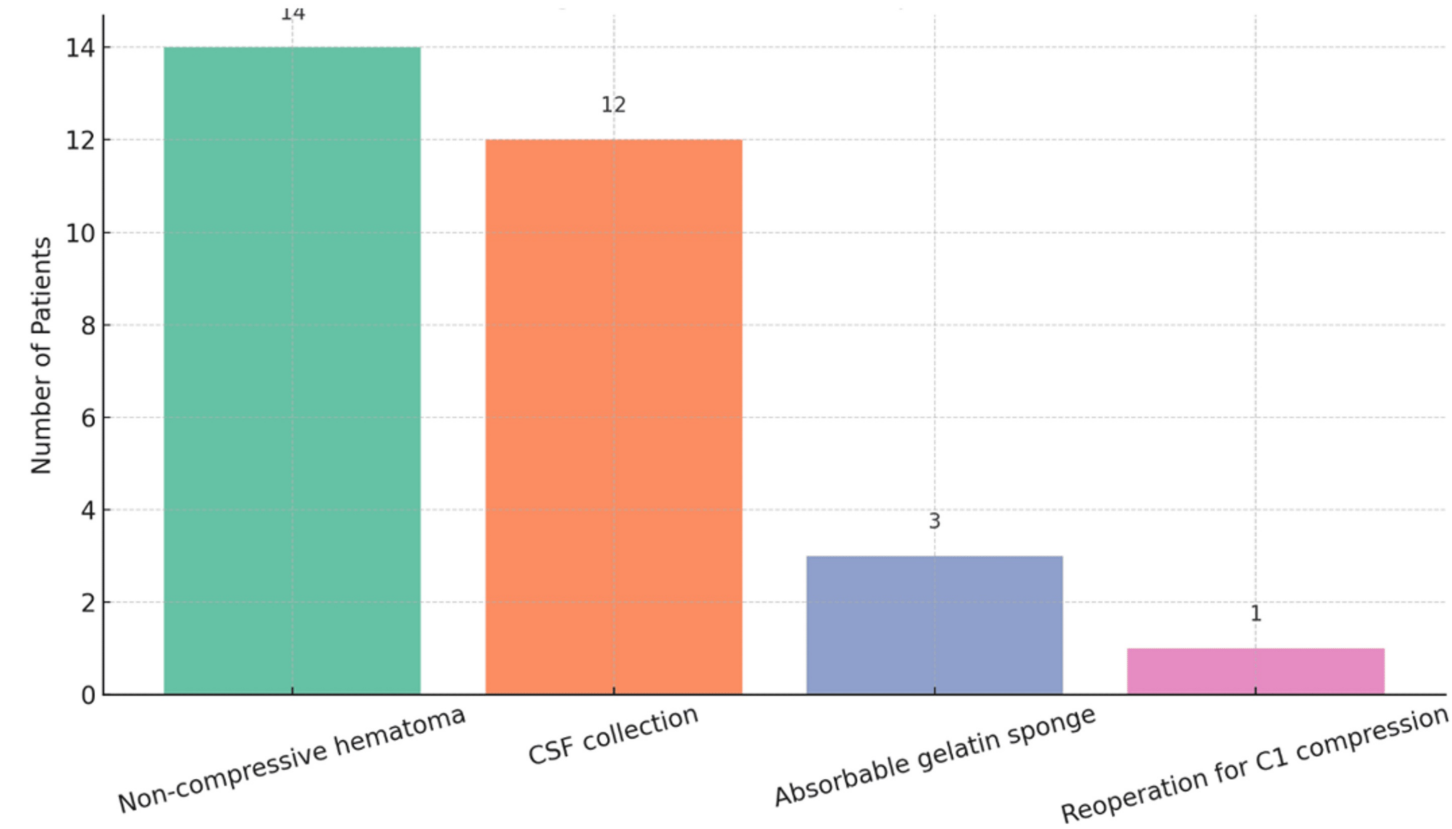
Graphical representation of immediate postoperative MRI findings.

Effacement of the CSF column was observed in seven patients on postoperative MRI. Of these, one had undergone anterior decompression (Figure 1), while the rest had posterior decompression with or without instrumentation. Preoperatively, three patients had severe myelopathy (mJOA < 11), two had moderate myelopathy (mJOA 11-14), and two had mJOA scores between 15 and 17. Radiologically, four patients had severe stenosis (Kang's Grade 3), two had Grade 2 stenosis, and one had Grade 1 stenosis. In four patients, no additional levels were decompressed, while in three, only one extra level, either above or below the stenosis, was addressed. 

**Figure 2:**
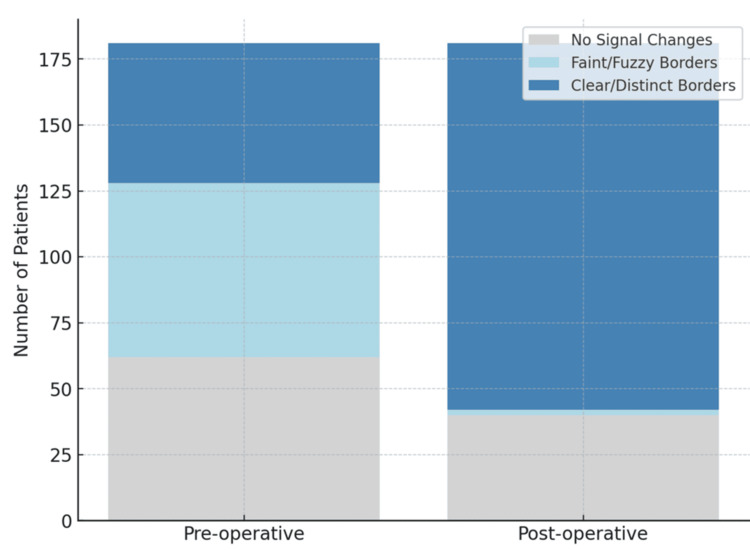
Stacked bar chart showing the distribution of intramedullary signal changes preoperatively and postoperatively.

**Figure 3:**
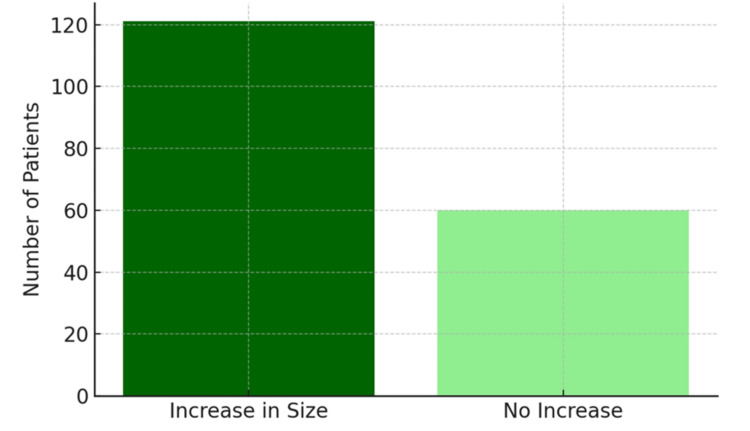
Bar chart showing the number of patients with increased size of intramedullary signal changes postoperatively.

Of the 181 patients, 66 showed intramedullary signal changes with faint, fuzzy borders; 53 had changes with clear, distinct borders; and 62 had no intramedullary changes. On postoperative MRI, 40 patients continued to show no signal changes, two had signal changes with faint, fuzzy borders, and the remaining 139 had changes with clear, distinct borders. These findings are illustrated in the stacked bar chart (Figure 2) and summarized in Table 1. An increase in the size of intramedullary hyperintensity was observed in 121 patients compared with preoperative imaging (Figure 3). This increase in the clarity of intramedullary signal changes was statistically significant, with a paired t-test showing p < 0.001. 

**Table 1:** Distribution of intramedullary signal changes on preoperative and postoperative MRI scans.

Condition	Preop MRI	Postop MRI
No signal changes	62 patients	40 patients
Signal changes with faint and fuzzy borders	66 patients	2 patients
Signal changes with clear and distinct borders	53 patients	139 patients
Increase in size of intramedullary hyperintensity	-	121 patients

MRI findings were correlated with patients’ postoperative neurological status using mJOA and Nurick scores. Notably, increased intramedullary signal intensity on immediate postoperative MRI did not, by itself, warrant further surgery; additional interventions were undertaken only in patients with clinical deterioration. This indicates that early postoperative MRI is most useful for detecting residual compression, missed levels, or acute complications, rather than predicting immediate neurological recovery.

Among patients with no intramedullary signal changes preoperatively (Grade 0), 12 developed Grade 2 changes postoperatively, while 2 developed Grade 1 changes. All patients with preoperative cord changes that had fuzzy borders developed distinct borders after surgery. None of the patients with preoperative Grade 2 changes showed regression in signal intensity. Fifty-six patients had severe myelopathy, defined by an mJOA score < 11. Of these, 36 had Kang’s Grade 3 stenosis and 20 had Grade 2 stenosis. Twenty patients showed no intramedullary signal changes, 19 had Grade 2 changes, and 17 had Grade 3 changes. After decompression, 42 patients had Grade 2 signal intensity changes, 1 had Grade 1 changes with fuzzy borders, and 13 showed no visible intramedullary signal changes.

One hundred six patients had moderate myelopathy (mJOA 11-14). Of these, 71 had Grade 3 stenosis on preoperative MRI, 32 had Grade 2, and 3 had Grade 1. Thirty-five patients showed no preoperative intramedullary signal changes, 39 had changes with indistinct borders, and 32 had Grade 2 signal changes. Postoperatively, 84 patients demonstrated Grade 2 signal changes, while 22 showed no changes on MRI. Non-compressive hematomas were observed in 14 patients, and CSF collections in 12. Absorbable gelatin sponge used for hemostasis was identified in three cases. These patients were managed conservatively by delaying drain removal, with discharge postponed until drain output decreased. Neurological status was closely monitored, and since no deterioration occurred, further imaging was not required. In one patient, immediate postoperative MRI revealed persistent compression at the C1 level following C2-C7 laminectomy, necessitating reoperation for adequate decompression. These findings are illustrated in Figure 1.

## Discussion

In patients undergoing posterior decompression, the average number of levels decompressed was about three, while postoperative MRI showed decompression at approximately four levels. Additional levels were typically included during posterior surgery to ensure adequate cord fallback. Effacement of the cervical spinal cord was observed in seven patients, occurring at the cranial or caudal edge of the decompression site, likely due to posterior migration of the cord after laminectomy. Four patients with postoperative residual stenosis had severe preoperative stenosis with visible intramedullary signal changes; two also showed cord deformation preoperatively. One patient demonstrated only effacement of the CSF column around the cord. In these cases, decompression was limited to the levels of stenosis identified preoperatively. All patients were advised routine follow-up, with careful neurological examination at each visit to allow early detection of new deficits.

**Figure 4:**
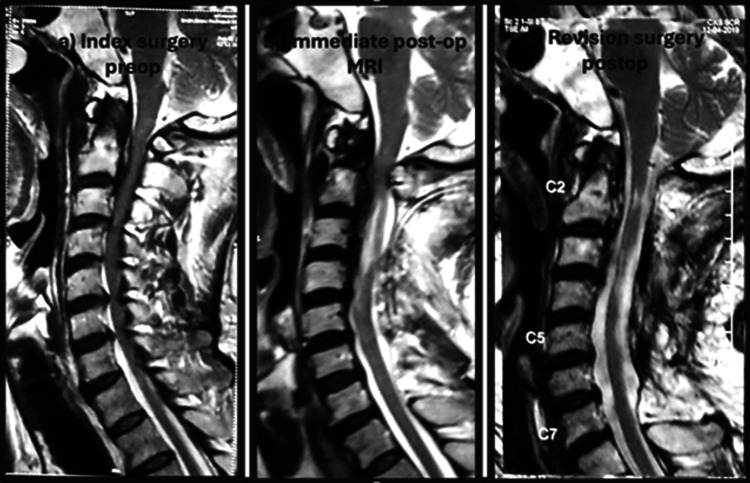
Images from the redo cervical laminectomy case. (A) Preoperative MRI showing stenosis at C2-C6 levels. (B) Immediate postoperative MRI after the index surgery demonstrating inadequate decompression (arrow). (C) Post-revision MRI following C1 arch excision showing adequate decompression.

In one case, a patient underwent a planned C2-C7 laminectomy; however, immediate postoperative MRI revealed persistent compression at C1 (Figure 4). Following the laminectomy, posterior compression occurred due to cord fallback and the increased size of the decompressed cord. The immediate postoperative MRI was therefore critical in identifying inadequate decompression secondary to cord fallback. The patient underwent reoperation the following day to achieve adequate decompression. A recent study by Shimizu et al. similarly reported that residual anterior cord compression after multilevel posterior decompression, particularly at cranial levels such as C1-C2, may result from insufficient initial decompression or cord fallback into the expanded dural space [8].

Joaquim et al. proposed the C3-C2 spinolaminar line test on lateral cervical radiographs as a simple and effective screening tool for detecting C1 stenosis [9]. This method, which identifies cases where the anterior lamina of C1 lies anterior to the extended C3-C2 spinolaminar line, may also have relevance in the postoperative assessment of patients undergoing multilevel laminectomy. In our setting, this test could be used preoperatively to anticipate cases in which cord fallback and expansion after C2-C7 decompression might lead to secondary compression at C1. Patients with such anatomical configurations may benefit from prophylactic C1 arch excision during the initial surgery to avoid residual or adjacent-level stenosis. However, this hypothesis requires confirmation through further prospective studies [9].

Rissanen et al. reported in their retrospective review that the most common cause of reoperation after cervical laminectomy without fusion was recurrent stenosis at the same or adjacent level [10]. Similarly, Bhalla et al. found that 10% of patients with cervical myelopathy experienced neurological decline within one year of decompression for CSM. In their series of 50 patients, those who deteriorated showed less than 1 mm of change in the anteroposterior cord diameter on MRI [11]. They also noted that inadequate decompression was more frequent in patients treated with anterior approaches than in those treated posteriorly. By contrast, in our series, most patients with inadequate decompression (n = 6/7, 85.7%) had undergone posterior decompression with or without fixation. The relatively high rate of inadequate decompression in their study led the authors to recommend routine postoperative MRI for cervical myelopathy. Flexion-extension MRI may further improve diagnostic yield in borderline cases, as Li et al. demonstrated instances of dynamic compression visible only on flexion or extension images when neutral MRI appeared satisfactory [12]. While spinal cord signal changes in degenerative cervical myelopathy are usually chronic, the transient increase in intramedullary signal seen on immediate postoperative MRI mainly aids in identifying residual stenosis, missed levels, or acute complications such as hematoma or CSF collections. Importantly, these imaging findings alone did not determine surgical management; reoperations were guided by clinical deterioration. Thus, the value of immediate postoperative MRI lies in confirming adequate decompression and enhancing patient safety, rather than predicting short-term neurological recovery.

Our results indicate that immediate postoperative MRI is sensitive for detecting residual cord compression and accurately delineates the number of decompressed levels. In our cohort, 121 patients demonstrated an increase in the size of intramedullary signal intensities, a statistically significant change. Sarkar et al. [13], in their study of 56 patients undergoing anterior decompression for CSM, reported preoperative intramedullary signal changes in 96% of cases, with three-fourths classified as Type 1 changes. Postoperatively, 71% of patients exhibited Type 2 intramedullary signal changes. Unlike our findings, their study noted a decrease in lesion size compared with preoperative imaging, and importantly, even patients with extensive intramedullary changes experienced meaningful clinical improvement. A key difference is that their postoperative MRIs were obtained at a median of 16 months, whereas our study focused on the immediate postoperative period. Notably, Abel et al. [14] observed that anterior decompression may promote greater regression of intramedullary T2 signal abnormalities than posterior approaches in OPLL patients, suggesting that surgical strategy may influence signal evolution. Taken together, these findings imply that the early postoperative increase in intramedullary signal changes we observed is likely transient and expected to regress with time.

Arvin et al. reported on patients with CSM who underwent MRI both preoperatively and six months postoperatively [15]. Their study investigated whether intramedullary signal changes at six months predicted neurological recovery and found that persistence of T2-weighted hyperintensity and lack of cord re-expansion correlated with poorer outcomes. Importantly, none of their patients demonstrated persistent cord compression on postoperative MRI. They observed signal changes in 13 patients: seven showed a transition from diffuse to focal signals, three from focal to diffuse, and three experienced complete resolution of intramedullary changes. In contrast, in our cohort, most patients demonstrated an increase in the size and intensity of intramedullary signal changes within the first 24 hours after surgery.

Harada and Mimatsu were the first to describe postoperative MRI changes after decompressive surgery for cervical myelopathy [16]. Using T1-weighted sagittal images, they focused on morphological changes in the cord but did not report intramedullary signal alterations, as T2-weighted images were not evaluated. Nagata et al. [17] later studied pre- and postoperative MRIs in 173 patients with cervical spondylotic myelopathy, assessing only midline sagittal images. Outcomes were categorized based on whether cord morphology improved or remained unchanged, and they observed a correlation between restoration of cord morphology at a mean follow-up of 1.5 years and clinical outcomes [16]. The only study to examine immediate postoperative MRI changes was published by Mastonardi et al. in 2007 [18]. They used an intraoperative scanner to perform MRI both before and immediately after anterior decompressive surgery for CSM, prior to the end of anesthesia. While none of their patients had residual compression on follow-up MRI at six months, very early imaging occasionally gave the false appearance of inadequate decompression due to the presence of the posterior longitudinal ligament, blood, or hemostatic materials. Additionally, four patients demonstrated regression of intramedullary signal changes on MRI scans performed immediately after surgery. However, this regression did not correlate with clinical outcomes on long-term follow-up. 

In our series, performing MRI after 24 hours of surgery yielded fewer false-positive findings of inadequate decompression. The presence of blood near the cord, which may mimic residual compression, was likely minimized by the routine use of drains in all patients. Intramedullary signal changes were also observed in a larger proportion of patients, and none showed regression of cord signal changes in the immediate postoperative period. A review of the literature indicates that most studies report regression in the size of intramedullary changes and transition to sharper borders within 15-45 days after surgery [19]. Our findings suggest that, at 24-48 hours after surgery, there is often a transient increase in cord signal changes, though the borders typically become more distinct during this early period. A better understanding of these signal evolution patterns may help distinguish reversible ischemia and edema from irreversible myelomalacia, particularly in the subacute setting [20].

Our results also show that routine postoperative screening MRI is effective in detecting residual stenosis and missed levels. This study adds to the understanding of how intramedullary signal changes evolve after decompression for cervical and dorsal myelopathy. The main limitation is the single time point of data collection; future studies should include serial imaging at multiple postoperative intervals to clarify the natural course of these changes. Additionally, the lack of long-term follow-up and correlation with neurological recovery prevents us from determining whether inadequate decompression in some patients led to deterioration. While intramedullary signal changes in degenerative cervical myelopathy are generally chronic, the transient increase seen on immediate postoperative MRI appears to serve mainly in identifying residual compression, missed levels, or acute complications such as hematoma or CSF collection. Importantly, these changes alone did not prompt reoperation; surgery was reserved for patients with clinical deterioration. Therefore, early postoperative MRI should be viewed primarily as a safety tool to confirm decompression adequacy and detect treatable complications, rather than as a predictor of neurological recovery.

## Conclusions

This study demonstrates that postoperative screening MRI is valuable in detecting residual compression, significant hematomas, and CSF collections, allowing timely precautions and interventions. It also offers medicolegal protection for surgeons while helping ensure optimal patient outcomes. A limited screening MRI can identify residual stenosis, as well as compressive and non-compressive hematomas, and can detect postoperative CSF collections that may indicate an unrecognized intraoperative durotomy. Early postoperative MRIs are typically characterized by an increase in the size and intensity of spinal cord signal changes.
